# Gender differences in health-related quality of life of adolescents with cystic fibrosis

**DOI:** 10.1186/1477-7525-4-5

**Published:** 2006-01-24

**Authors:** Renata Arrington-Sanders, Michael S Yi, Joel Tsevat, Robert W Wilmott, Joseph M Mrus, Maria T Britto

**Affiliations:** 1 Adolescent Medicine Fellow, Division of General Pediatrics and Adolescent Medicine, The Johns Hopkins University, Baltimore, Maryland, USA; 2 Division of General Internal Medicine, University of Cincinnati, Cincinnati Ohio, USA; 3 Veterans Healthcare System of Ohio (VISN 10), Cincinnati, Ohio, USA; 4 Institute for the Study of Health, University of Cincinnati, Cincinnati, Ohio, USA; 5 Division of Adolescent Medicine, Cincinnati Children's Hospital Medical Center, Cincinnati, Ohio, USA; 6 Department of Pediatrics, Saint Louis University, St. Louis, Missouri, USA; 7 GlaxoSmithKline, Research Triangle Park, NC, USA

## Abstract

**Background:**

Female patients with cystic fibrosis (CF) have consistently poorer survival rates than males across all ages. To determine if gender differences exist in health-related quality of life (HRQOL) of adolescent patients with CF, we performed a cross-section analysis of CF patients recruited from 2 medical centers in 2 cities during 1997–2001.

**Methods:**

We used the 87-item child self-report form of the Child Health Questionnaire to measure 12 health domains. Data was also collected on age and forced expiratory volume in 1 second (FEV_1_). We analyzed data from 98 subjects and performed univariate analyses and linear regression or ordinal logistic regression for multivariable analyses.

**Results:**

The mean (SD) age was 14.6 (2.5) years; 50 (51.0%) were female; and mean FEV_1 _was 71.6% (25.6%) of predicted. There were no statistically significant gender differences in age or FEV_1_. In univariate analyses, females reported significantly poorer HRQOL in 5 of the 12 domains. In multivariable analyses controlling for FEV_1 _and age, we found that female gender was associated with significantly lower global health (p < 0.05), mental health (p < 0.01), and general health perceptions (p < 0.05) scores.

**Conclusion:**

Further research will need to focus on the causes of these differences in HRQOL and on potential interventions to improve HRQOL of adolescent patients with CF.

## Background

Cystic fibrosis (CF) is a genetic disorder that affects multiple organ systems. Treatments have improved overall survival so that those born today have a median life expectancy of approximately 40 years [1]. Much of the morbidity and mortality associated with cystic fibrosis is due to pulmonary disease, and investigations have shown that early, aggressive, and center-based care improves prognosis [2]. Objective clinical parameters, such as aerobic fitness, pulmonary function, nutritional status, and aggressive treatment with antibiotics, are associated with improved health outcomes [2]. In the past, clinical morbidity and mortality rates in children and adolescents were monitored as primary outcomes [3], but more recently, to provide the scientific basis for practice guidelines and to measure treatment effectiveness from patients' perspectives, there has been increasing focus on the examination of health-related quality of life (HRQOL) [4,5], Thus, the Cystic Fibrosis Foundation and the National Heart, Lung, and Blood Institute recommend incorporating quality of life as an outcome measure for patients with CF [6]. In CF populations, the measurement of HRQOL is one approach that has been used to correlate clinical outcomes and the impact of both disease progression and treatments from patients' perspectives [7-9].

Many factors have been shown to influence survival in patients with CF. Biologic and physiologic factors such as pancreatic insufficiency and pseudomonas infections, and socioeconomic factors such as inadequate or no health insurance are associated with decreased survival rates [2,10,11]. Britto found that, compared with the general population, patients with CF report lower physical health scores, independent of lung function, nutritional status or demographic factors [12]. Moreover, female patients with CF have been shown across age strata to have a shorter life expectancy, more serious respiratory infections, and *Pseudomonas aeruginosa *infections at a younger age than male patients, although the etiology of gender differences is unclear [13-15]. Also, higher health values, including higher time tradeoff (TTO) scores, have been linked to male gender in patients with CF [16].

In other populations, studies have shown that gender plays a part in the way patients – including adolescents – perceive their own HRQOL and health status [17-21,30]. Adult female patients with chronic illnesses have reported poorer HRQOL than males in prior studies [22-25]. Evidence suggests that, in general, men and women may respond differently to poor health and women may, in turn, report poorer general health than men [26-30]. This difference has also been seen among adolescents. In one European study, adolescent females reported lower HRQOL with regard to physical health, mood, and self perception than adolescent males. [31] This difference was noted to have developed around age 12 and persisted through late adolescence.

Few studies have examined how the gender of patients with CF may impact their HRQOL when accounting for the severity of illness [32-35]. With this investigation, we wanted to determine if gender differences exist in self-reported HRQOL in adolescents with CF. Our specific objectives were: 1) to determine if there are gender differences in HRQOL in adolescent patients with CF; and 2) to determine which HRQOL domains are associated with differences when stratified by gender, after controlling for markers of disease severity. We hypothesized that gender differences exist, with female adolescents reporting poorer HRQOL than males.

## Methods

### Study design

We performed analyses of data from two previously published cross-sectional questionnaire studies of adolescent outpatients with CF [12,16]. The combined datasets were used to evaluate for gender differences in adolescents with CF.

### Recruitment and data collection

In each of the prior studies, subjects previously diagnosed with CF were recruited from Cincinnati Children's Hospital Medical Center in Cincinnati, Ohio and The Children's Medical Center in Dayton, Ohio between 1997–2001. Patients and their families were invited to participate in each of the prior studies by either a physician or a research coordinator at the time of their regularly scheduled quarterly clinic appointment, by telephone, or by mail. Patients in each study were recruited and completed the questionnaire in the same manner. Patients between 10 and 18 years of age were recruited, and we excluded patients with CF who had undergone lung transplantation. Informed consent was obtained from the patient. If the patient was younger than 18 years, informed consent was obtained from the parent or guardian and assent was obtained from the patient. The study was approved by the institutional review boards at both participating sites.

### Health-related quality of life measures

The Child Health Questionnaire (CHQ) was used to measure the adolescent's HRQOL in each of the prior studies [36]. The CHQ is a generic health status instrument that has been validated, used in a number of populations with chronic illness, including adolescents with CF, and found to be reliable [12,16,37-39]. The Child Form-87 (CF-87) of the CHQ was designed to measure 12 health domains: global health – subjective overall health; physical functioning – physical limitations due to health-related problems; bodily pain – intensity and frequency of general pain and discomfort; behavior – aggression, delinquency, hyperactivity/impulsivity and social withdrawal; mental health – anxiety, depression and positive affect; self esteem – satisfaction with school and athletic ability, looks/appearance, ability to get along with others and family, and life overall; general health perceptions – subjective assessment of past, future and current health and resistance/susceptibility to sickness; family cohesion – how well the patient's family gets along with one another; family activities – frequency of family activities; role limitations due to physical, emotional, and behavioral problems; and change in health in the past year. On each subscale, a score of 0 represents worst functioning and 100 represents best functioning, except for a change in health, where "1" represents health much worse than 1 year ago, "2" represents health somewhat worse than 1 year ago, "3" represents health about the same as 1 year ago, "4" represents health somewhat better than 1 year ago, and "5" represents health much better than 1 year ago. The highest possible score indicates the absence of a negative state, whereas lower scores indicate greater limitations in the HRQOL domain.

### Clinical measures

Data were collected on age, gender, and forced expiratory volume in 1 second (FEV_1_). Pulmonary function and exacerbations have been associated with differences in HRQOL in adults and older adolescents [12]. Pulmonary function was determined by using spirometry and total body plethysmography according to American Thoracic Society Standards (American Thoracic Society 1991). Demographic data and clinical data were obtained from the CHQ or clinic chart review.

### Statistical analysis

All analysis were performed with SAS software, version 8.0 (SAS Institute, Inc, Cary, NC). Comparisons between continuous variables were conducted using two-tailed t tests and comparisons between categorical variables were conducted using χ^2 ^tests. We used multivariable linear regression analysis to determine if the independent variable (gender) was associated with the outcome variable (each CHQ subscale), controlling for FEV_1 _and age. Because of the categorical nature of the GH outcome variable, we performed multivariable analysis by using ordinal logistic regression. Each variable was entered into multivariable linear regression if in univariate analysis it had a significance level equal to or greater than 0.20. Because some the variables were not normally distributed, to assess the robustness of our results, we repeated all of the comparisons using non-parametric methods and the results were qualitatively the same. We found the parametric univariate comparisons to be consistent with the parametric multivariable methods. We also performed the regressions with the outcome log transformed and the results were qualitatively identical. The non-transformed results are presented for ease of interpretation.

Although there is currently no "gold standard" or consensus by which the clinical importance of differences HRQOL scores can be determined, methods have been proposed to estimate clinically important differences (CID) in HRQOL [40-43]. In one approach, the minimal clinically important difference [MCID] is defined by an effect size of >0.20, where effect size = [mean_M_-mean_F_]/[SD_M_] [40]. In this schema, effect sizes of 0.20–0.49 indicate 'small' effect sizes, 0.50–0.79 indicate 'moderate' effect sizes, and >0.80 indicate 'large' effect sizes. We used effect sizes of differences in HRQOL between males (M) and females (F) to assess for clinically important differences.

## Results

Ninety-eight adolescents aged 10–18 years completed the CF-87 (Table 1). The mean (SD) age was 14.6 (2.5) years; 50 (51.0%) were female; and the mean (SD) FEV_1 _was 71.6% (25.6%) of predicted. There were no significant differences between males and females for age or FEV_1_. Table 2 summarizes the mean, SD, range, and percent at the floor and ceiling for each scale.

**Table 1:** Characteristics of Participants

**Gender**	**Patients, # (%)**	**Age, yr (SD)**	**FEV_1_, %_predicted_(SD)**
Female	50 (51%)	14.5 (2.8)	67 (26)
Male	48 (49%)	14.6 (2.2)	76 (25)
P-Value		0.80	0.07

**Table 2:** Health-related quality of life by gender

**Domain**	**Mean (SD)**	**Median**	**Interquartile range (IQR)**	
**Females**				
				
Global Health*	65 (23)	60	60	85
Physical Functioning*	86 (17)	92.6	81.5	100
Bodily Pain	67 (24)	70	50	80
Behavior*	79 (15)	83.6	72	88
Mental Health*	70 (19)	76.6	61.7	85.2
Self-esteem*	77 (16)	78.6	66.1	89.3
General Health Perception*	49 (20)	49.8	34.6	63.3
Family Cohesion*	74 (23)	85	60	85
Family Activities*	71 (21)	75	50	87.5
Role/social Emotional	86 (18)	100	77.8	100
Role/social Physical	92 (18)	100	66.7	100
Role/social Behavioral	87 (20)	100	100	100
Change in Health	3.6 (1.2)	4.0	3.0	5.0

**Males**				
				
Global Health*	79 (24)	85	60	100
Physical Functioning*	91 (14)	100	87.0	100
Bodily Pain	71 (22)	80	60	80
Behavior*	84 (11)	87.1	78.5	92.2
Mental Health*	80 (13)	82.8	70.3	90.3
Self-esteem*	82 (18)	87.5	67.9	96.4
General Health Perception*	62 (21)	61.3	48.3	77.9
Family Cohesion*	81 (16)	85	60	92.5
Family Activities*	80 (21)	83.3	70.8	95.8
Role/social Emotional	90 (20)	100	88.9	100
Role/social Physical	90 (21)	100	88.9	100
Role/social Behavioral	91 (19)	100	94.4	100
Change in Health	3.7 (1.0)	4	3	5

*P value < 0.05 in univariate analysis comparing males and females

### Health status

**Figure 1:**
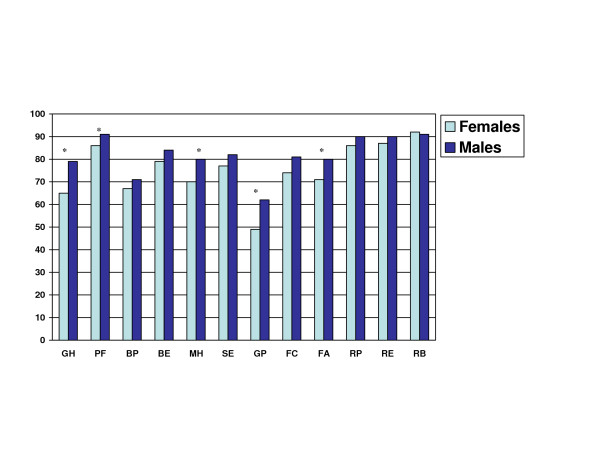
**Health-Related Quality of Life by Domain and Gender**. Health scores ranging from 0–100 are shown on the Y-axis and domains on the X-axis. The asterix (*) indicates p-value < 0.05 for the difference between males and females. **Domains**: Global Health (GH); Physical Functioning (PF); Bodily Pain (BP); Behavior (BE); Mental Health (MH); Self-esteem (SE); General Health Perception (GP); Family Cohesion (FC); Family Activities (FA); Role/social Physical; Role/social Emotional (RE); Role/social Behavioral (RB).

Female adolescents with CF reported poorer HRQOL than males in all 12 of the domains except for the role behavioral domain (Table 2). In univariate analysis, females reported significantly poorer health in the global health, physical functioning, mental health, general health perceptions, and family activity domains (p < 0.05; Figure 1; Table 2). There were no significant differences noted between males and females for the change in health domain.

**Figure 2:**
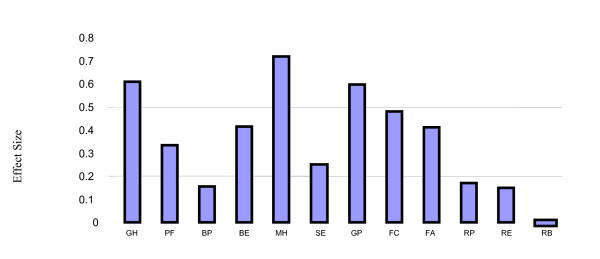
**Effect Sizes for Gender Differences in HRQOL**. The domains of HRQOL are shown on the X-axis and the effect size on the Y-axis where effect size = [mean_M_-mean_F_]/SD_M_. Effect sizes between 0.2 and 0.5 are felt to be minimally clinically important and those between 0.5 and 0.8 are felt to be moderately clinically important. Positive effect sizes indicate better HRQOL for males than females. Role physical (RP) domain is negative because males reported worse HRQOL than females. **Domains**: Global Health (GH); Physical Functioning (PF); Bodily Pain (BP); Behavior (BE); Mental Health (MH); Self-esteem (SE); General Health Perception (GP); Family Cohesion (FC); Family Activities (FA); Role/social Physical; Role/social Emotional (RE); Role/social Behavioral (RB).

Three health domains of the CHQ showing statistically significant gender differences in the multivariate models demonstrate an effect size that is moderately clinically important between males and females: the global health, mental health and general health perception scales (Figure 2). The difference in role physical domain was negative because males reported poorer HRQOL in the role physical domain than females.

In multivariable analyses controlling for age and FEV1, females reported poorer global health, mental health, and general health perceptions (p < 0.05; Table 3). Gender was no longer significantly associated with physical functioning and family activities when controlling for age and FEV1.

**Table 3:** Domains with significant gender differences in multivariable models

	**β-Coefficient**	**P value**	**R^2^**
Global Health (GH)	1.1	<0.05	0.44*
Mental Health (MH)	10.4	<0.01	0.09
General Health Perception (GP)	9.7	<0.05	0.21

* Somers' D, used for logistic procedure; multivariable model controlling for age and FEV_1_

## Discussion

Female patients with CF have been shown to have much shorter life expectancies than males with CF, with a median decrease of 4 years until the age of 20 [14,44]. We sought to determine if gender differences exist for HRQOL for adolescent patients with CF.

In our cohort of adolescents with CF, significant gender differences in HRQOL existed between males and females. On average, female adolescents with CF scored lower on all health domains with the exception of the role behavior domain. Even when controlling for age and lung disease severity, female adolescents had significantly lower scores in mental health, global health, and perceptions of general health. Physical functioning was not related to gender when controlling for age and lung disease severity. This may indicate that age and disease severity is more important than gender in predicting physical aspects of HRQOL. In addition, the level of family activities, which may also be influenced by age of the patient and severity of lung disease, were no longer significant when controlling for those factors.

Previous research has found that female adolescents with CF have a more accurate perception of objective clinical health status than male adolescents [26], but rely more on denial to cope [45]. Such negative ways of coping can result in poor correlation between pulmonary function and general health perceptions. When compared to adolescent males, adolescent women, have also been found to use more of both positive and negative coping strategies to withstand pain [34,46,47]. Avoidance strategies may be viewed as 'negative' or maladaptive by health professionals if it means that patients avoid doing their treatments, but from the perspective of patients may be psychologically adaptive as a means to escape the world of CF for a while. Additionally, some studies have found that adolescent females report more physical health symptoms, psychological symptoms and use more emotion-oriented and problem oriented coping strategies than adolescent males. [48] Because coping styles may be associated with medication adherence, gender differences and coping skills need to be considered when correlating clinical markers with changes in HRQOL.

From a developmental psychology perspective, changes in HRQOL may be explained by hormonal development as well as a physical and social transition from childhood to adulthood. As adolescents transition, peer and social groups emerge, cognitive abilities develop, and they become more independent from their families. This stage of development may affect perceived physical health. Some have described that perceived health is determined by personal factors such as gender, school achievement, drug use, health behaviors, and the adolescents' environment, such as peer/parent relationships and the family's income [49]. Others have linked risk-taking behaviors to lower family income [50]. However, some data suggest that gender differences play a more significant role than class differences with regard to differences in health [51]. Similar to studies done in non-CF populations [31], we found gender differences among adolescents with CF in the areas of mental health and general health perceptions. Differences in physical functioning did not persist in this study possibly because body image also contributes to one's perception of physical health and physical functioning is linked to pulmonary function. Studies have shown little difference in body image between patients with CF and healthy age-matched controls [52]. Females more often overestimate their weight, while males underestimate their weight. A significantly greater proportion of males than females perceived their weight status as underweight and therefore, were more likely to take enteral supplements [53]. Willis and colleagues [54], found that young men and women with CF identify first as young men and women, rather than adults with CF. This combined with social desires to stay slender, has been suggested to contribute to non-adherence.

Powers [55] found that adolescents reported strong relationships between pulmonary function and self-reports of pain, general health, and school-activity limitations, but pulmonary function was not related to physical functioning, emotional or behavioral health. Perhaps because gender differences factor into global health, physical function, and general health perception among adolescents, such a relationship with pulmonary function is not established. In this study, female adolescents with CF reported poorer mental health, global health, and general health perceptions. Such gender differences might limit associations between pulmonary function and certain health domains when the effect of gender is not taken into account.

There are some limitations of this study. Although we recruited patients from 2 CF centers, the sample size was relatively small. A few of the health domains exhibited ceiling effects. The CHQ is a generic questionnaire used to measure HRQOL in diverse pediatric populations and may not have been able to detect small differences in HRQOL in patients with CF. Accordingly, the generalizability of our findings is uncertain. Because we examined HRQOL in two prior completed studies, we were unable to examine HRQOL differences in those who refused and those who agreed to participate. Patients with more severe disease (with greater likelihood of being females) may have participated in the study at disproportionately low rates when compared with healthier counterparts. For example, in our population, the overall FEV_1 _was reflective of mild disease. Clinical variables, such as *pseudomonal *infection, nutrition status, and history of co morbidities such as diabetes, and social variables such as socioeconomic or insurance status, were not available for many observations and thus were not controlled for, which may partially explain the HRQOL differences we found between genders.

Multiple studies have shown that females, including adolescents, report poorer HRQOL than males [8,18,20,56], often despite having similar objective clinical  measures like pulmonary function. It is possible that such differences were based on perception of health, rather than actual health status. Females had poorer HRQOL in primarily the psychological domains of health, when controlling for disease severity and age. The psychological domains of health may potentially affect therapy and should be considered when treating female adolescents with cystic fibrosis. Other social constructs that affect adolescent skill building, including self-construct, self-efficacy, identity formation and social support may be responsible for overall health perceptions in adolescent males and females.

## Conclusion

Gender differences in HRQOL appear to exist between adolescent males and females with CF. Further research will need to focus on the causes of such gender differences in HRQOL and on potential interventions to improve HRQOL for all adolescents living with CF.

## Competing interests

The author(s) declare they have no competing interests.

## Authors' contributions

RA, MY, JT, RW, JM and MB worked on the conception and design. Acquisition of data was performed by MY and MB. Analysis and interpretation of data was performed by RA, MY, JM and MB. The manuscript was drafted by RA, MY and MB. RA, MY, JT, JM, RW and MB were involved in critical revision of the manuscript for important intellectual content. Statistical analysis performed by RA, MY, and JM. MY, JT, JM, RW and MB provided administrative, technical, or material support. Study supervision was performed by MY, JT, RW and MB.
